# Hernia of Umbilical Cord with Congenital Short Gut

**Published:** 2014-04-01

**Authors:** Bilal Mirza, Muhammad Saleem

**Affiliations:** Department of Pediatric Surgery, The Children’s Hospital and the Institute of Child Health Lahore, Pakistan

**Figure:**
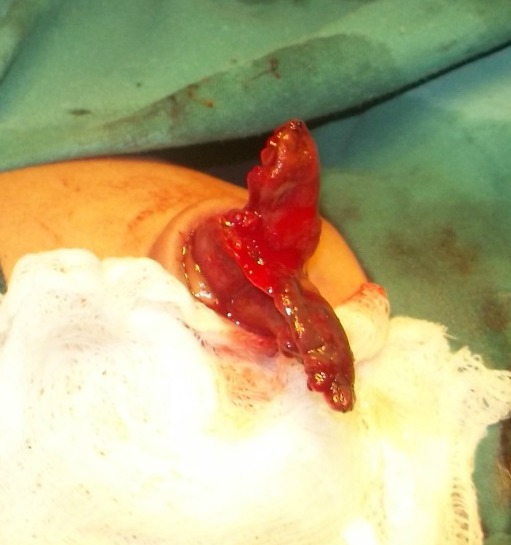
Figure 1: Bowel loops after removing sac of hernia of umbilical cord

**Figure:**
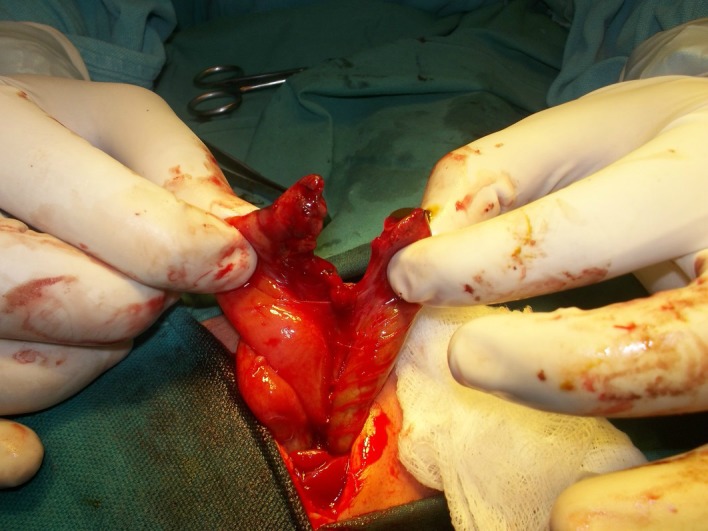
Figure 2: 7cm of jejunum (jejunal atresia) and 6cm of sigmoid colon (colonic atresia)

A 1-day-old male baby, weighing 2kg, presented with bilious vomiting and a mass at umbilicus covered by abnormally thickened base of umbilicus (hernia of umbilical cord). The mother was not on any antenatal follow-up. The delivery was spontaneous vaginal at home. On examination abdomen was scaphoid. Abdominal radiograph showed three air fluid levels indicative of associated small bowel atresia. At operation, two blind ending loops of the bowel were found after removing the sac of the hernia of umbilical cord. Further exploration revealed congenital short gut. There was only 7cm of the jejunum and 6cm of the sigmoid colon present in the abdominal cavity. Jejunocolic anastomosis performed. Patient remained admit for two weeks on TPN and then parents took the child against medical advice.

Hernia of umbilical cord is a well-known entity to the pediatric surgeons. It is herniation of the small bowel into the base of the umbilicus. Rest of the anterior abdominal wall is usually normal in these cases. Embryologically, there is physiological herniation of the mid gut into the umbilical coelom during 5-6th week of gestation. The herniated gut then returned during 10-12th week of gestation to the abdominal cavity. It is that time when a small part of mid gut could not return completely into the abdomen and thus retained in the base of umbilicus resulting in hernia of umbilical cord.[1-3] Our case was unique as on removing the sac, there were jejunal and colonic atresia with only 15cm of total intestinal length from duodeno-jejunal (DJ) junction to the rectum. It can be speculated that an in-utero volvulus of the mid gut during physiological herniation phase might have resulted in intestinal loss and resorption resulting in entry and exit levels atresia. Haas et al,[1] followed 8 cases of hernia of umbilical cord; one of 8 developed progressive dilatation of the contents of the hernia of umbilical cord resulting is rupture of the umbilicus and fetal demise. In our case, the second possibility could be the rupture of the umbilical hernial sac resulting in evisceration of the bowel into the amniotic cavity where mid gut volvulus might have resulted in the intestinal loss and entry and exit level atresia. 

## Footnotes

**Source of Support:** Nil

**Conflict of Interest:** The author is an Editor of the journal. But he did not take part in the evaluation or decision making of this manuscript. The manuscript has been independently handled by two other editors

